# A Multiscale Analysis on the Superelasticity Behavior of Architected Shape Memory Alloy Materials

**DOI:** 10.3390/ma11091746

**Published:** 2018-09-17

**Authors:** Rui Xu, Céline Bouby, Hamid Zahrouni, Tarak Ben Zineb, Heng Hu, Michel Potier-Ferry

**Affiliations:** 1 Laboratory of Excellence on Design of Alloy Metals for Low-Mass Structure (Labex-DAMAS), Université de Lorraine, 57070 Metz, France; ruixu@whu.edu.cn (R.X.); tarak.ben-zineb@univ-lorraine.fr (T.B.Z.); michel.potier-ferry@univ-lorraine.fr (M.P.-F.); 2 School of Civil Engineering, Wuhan University, 8 South Road of East Lake, Wuchang, 430072 Wuhan, China; huheng@whu.edu.cn; 3 Université de Lorraine, CNRS, Arts et Métiers ParisTech, LEM3, F-57000 Metz, France; 4 Université de Lorraine, CNRS, Arts et Métiers ParisTech, LEM3, F-54000 Nancy, France; Celine.Bouby@univ-lorraine.fr

**Keywords:** shape memory alloys, architected cellular material, numerical homogenization, multiscale finite element method

## Abstract

In this paper, the superelasticity effects of architected shape memory alloys (SMAs) are focused on by using a multiscale approach. Firstly, a parametric analysis at the cellular level with a series of representative volume elements (RVEs) is carried out to predict the relations between the void fraction, the total stiffness, the hysteresis effect and the mass of the SMAs. The superelasticity effects of the architected SMAs are modeled by the thermomechanical constitutive model proposed by Chemisky et al. 2011. Secondly, the structural responses of the architected SMAs are studied by the multilevel finite element method (FE${}^{2}$), which uses the effective constitutive behavior of the RVE to represent the behavior of the macroscopic structure. This approach can truly couple the responses of both the RVE level and structural level by the real-time information interactions between two levels. Through a three point bending test, it is observed that the structure inherits the strong nonlinear responses—both the hysteresis effect and the superelasticity—of the architected SMAs at the cellular level. Furthermore, the influence of the void fraction at the RVE level to the materials’ structural responses can be more specifically and directly described, instead of using an RVE to predict at the microscopic level. Thus, this work could be referred to for optimizing the stiffness, the hysteresis effect and the mass of architected SMA structures and extended for possible advanced applications.

## 1. Introduction

Cellular materials are widely used for their high strength-to-weight ratio and high energy absorption performance (Gibson and Ashby [1]; Ashby et al. [2]). For instance, honeycomb, folded cellular materials and foam are usually used as the core of the sandwich structures for dissipating the kinetic energy, damping or reducing the weight of the structure (Ashby et al. [2]; Yazdani et al. [3]; Garcia-Moreno [4]; Hangai et al. [5]; Strano et al. [6]). However, honeycomb and folded cellular materials have high manufacturing costs and moisture problem, as well as buckling problems (Rashed et al. [7]). The mechanical behaviors of foams are too difficult to be accurately measured for their stochastic cells, which always results in the excessive use of materials to satisfy the safety factor. To overcome these shortcomings, partially-ordered foams allowing limited structural control of the pore and spatial distribution of pore levels, such as metal syntactic foams (Taherishargh et al. [8,9,10]; Broxtermann et al. [11]; Linul et al. [12]; Luong et al. [13]) are studied. Furthermore, architected cellular materials with an ordered structure were designed and studied during these years (Rashed et al. [7]; Pingle et al. [14]; Schaedler et al. [15]; Schaedler and Carter [16]; Lehmhus et al. [17]). Thanks to the highly developed additive manufacturing techniques, such as 3D printing (Ngo et al. [18]; Mostafaei et al. [19]) and selective laser welding (Mehrpouya et al. [20]; Rashed et al. [7]), the manufacturing of architected cellular materials is no longer impossible. Users can design a cellular material with a certain behavior by tuning its cellular parameters, such as the geometry, components, local mechanical properties, etc.

Architected cellular materials’ functionality could be extend by combining the features of various materials, such as shape memory alloys. It is well known that shape memory alloys, such as NiTi, can endure large deformation and recover their initial shape after unloading (see for example the reviews of Lagoudas [21], Patoor et al. [22], Lagoudas et al. [23], Tobushi et al. [24] and Cisse et al. [25]). This superelasticity effect brings high performance to SMA in energy absorption. When the given load reaches a critical level in a superelastic test, SMA will apparently soften due to its inner phase transformation. This behavior enables SMA to absorb the external energy as much as possible and prevents material from crushing or buckling. Such a kind of response is very similar to the ideal response of the cellular material designed by Schaedler et al. [15]. Meanwhile, the hysteresis effects of SMA can dissipate a large amount of energy. All mentioned features of SMAs meet the requirements of an architected structure for energy absorption applications very well. In addition, taking advantage of the lightweight and shape memory effect, architected SMAs may be designed for advanced applications in aerospace, civil engineering, etc.

To investigate the behavior of architected cellular SMAs, rare, but valuable works have been proposed (Machado et al. [26]; Ravari et al. [27]; Ashrafi et al. [28]). Machado et al. [26] proposed an experimental and modeling study on the cellular NiTi tube-based materials. In order to design and optimize the architected SMA tube materials, the authors studied the effective behavior of the thin-walled NiTi cellular materials by carrying out a study based on experiment and numerical simulation. The influences of SMAs’ material properties and cellular architecture on the effective behavior were investigated. To reduce the high cost of fabrication, Ravari et al. [27] focused mainly on numerical modeling for designing and optimizing SMA cellular lattice structures. The effects of the geometry and cellular imperfections on the effective behavior of the material were investigated by unit cell and multi-cell methods. This work was later developed by Ashrafi et al. [28], who proposed an efficient unit cell model with modified boundary conditions for SMA cellular lattice structures. The shape memory effect was also simulated by this model, which had good agreement with the experiment.

The above works mainly focused on the effective cellular response in order to represent or predict the behavior of architected SMA structures. Considering the scale separation between the microscopic cellular scale and macroscopic structural scale, however, it is difficult to predict the structural response of a unit cell without certain assumed boundary conditions, because the stress-strain states of the macroscopic structure are usually not uniform and the deformations at the microscopic level could be totally different. Thus, in order to directly simulate the structural responses of architected SMA, more appropriate numerical methods should be used. During the past few decades, multiscale modeling approaches have been developed and widely used (Kanoute et al. [29]; Geers et al. [30]; El Hachemi et al. [31]; Kinvi-Dossou et al. [32]). As one of the most popular and effective multiscale methods, the multilevel finite element method (FE${}^{2}$, see Feyel [33]) to describe the response of high nonlinear structures using generalized continua shows good performance in various applications, such as fiber buckling (Nezamabadi et al. [34]), composite shells (Cong et al. [35]), rate-dependent response (Tikarrouchine et al. [36]) and SMA-based fiber/matrix composites (Kohlhaas and Klinkel [37]; Chatzigeorgiou et al. [38]; Xu et al. [39]). In this approach, both the structural level and the RVE level are simulated by the finite element method (FEM). Two levels are fully coupled and computed simultaneously, where the unknown constitutive behaviors on the structure level are represented by the effective behaviors of homogenized RVEs, and the strain states of the RVEs are given by the associated integration points. The FE${}^{2}$ method shows good performance on multiscale modeling of SMA-based materials. Xu et al. [39] proposed a 3D FE${}^{2}$ model for simulating composites with stiff SMA fibers embedded in a soft matrix. This model was validated by the literature and showed a good ability in modeling the superelasticity and the shape memory effects of SMA composites.

Following previous works, it is therefore necessary and feasible to investigate the behavior of architected SMAs with a multilevel finite element model. Towards a better understanding of the studied architected SMA, unit cells (RVEs) with different void fractions are introduced to study the superelasticity effect of the materials and structures. In Section 2, a constitutive model for SMA is introduced briefly. Then, a parametric analysis with different void fractions at the cellular level is performed to predict the relations between the void fraction, the total stiffness, the hysteresis effect and the mass of the architected cellular SMAs. In Section 3, after a short presentation for the multilevel finite element model, multiscale modelings on architected SMA structures are carried out to simulate the structural and the cellular responses simultaneously.

## 2. Cellular Response

To investigate the architected SMA structures, RVEs with different geometrical parameters are studied firstly, then the structural response is investigated further by the computational homogenization method. First of all, a short presentation of the SMA constitutive model is given in the next subsection.

### 2.1. SMA Constitutive Model

The SMA model, proposed by Chemisky et al. [40], is adopted for the thermomechanical behavior modeling of the architected SMA. This model follows the work of Peultier et al. [41], who proposed a macroscopic phenomenological SMA approach based on the Gibbs free energy. This model was implemented on ABAQUS via user-defined materials (UMAT ) and validated by experiments. It was later improved by Chemisky and Duval; see Chemisky et al. [40]; Duval et al. [42]. Here, only a brief introduction is presented for the readers due to the limited length of the paper.

The SMA constitutive model used here is able to describe four different strain mechanisms: the elastic strain $\varepsilon^{e}$, the thermal expansion strain $\varepsilon^{th}$, the martensitic transformation strain $\varepsilon^{tr}$ and the twin accommodation strain $\varepsilon^{tw}$. To this end, the total strain is decomposed in the following form:

(1) $$\varepsilon =\varepsilon^{e}+\varepsilon^{th}+\varepsilon^{tr}+\varepsilon^{tw},$$

where the above strains are formulated as:

(2) $$\left\{\begin{array}{c} \varepsilon^{e}=S:\sigma ,\\ \varepsilon^{th}=\alpha \left(T-T_{ref}\right),\\ \varepsilon^{tr}=f{\tilde{\varepsilon }}^{tr},\\ \varepsilon^{tw}=f^{FA}{\tilde{\varepsilon }}^{tw}. \end{array}\right.$$

where S denotes the isotropic fourth order compliance tensor, α represents the isotropic thermal expansion tensor, $T_{ref}$ gives the temperature without expansion strain, *f* gives the martensitic volume fraction in SMA, the transformation strain ${\tilde{\varepsilon }}^{tr}$ describes the mean strain over the martensite related to martensite reorientation and the mean strain ${\tilde{\varepsilon }}^{tw}$ and self-accommodated martensitic volume fraction $f^{FA}$ are introduced to describe the twin accommodation over the martensite.

The potential energy of the SMA model is based on the following Gibbs free energy expression:

(3) $$\begin{array}{cc} G & =\left(U^{A}-TS^{A}\right)\left(1-f\right)+\left(U^{M}-TS^{M}\right)f-\frac{1}{2}\sigma :S:\sigma -\sigma :\alpha \Delta T-\sigma :{\tilde{\varepsilon }}^{tr}f-\sigma :{\tilde{\varepsilon }}^{tw}f^{FA}\\ & \;+\frac{1}{2}fH_{tr}{\tilde{\varepsilon }}^{tr}:{\tilde{\varepsilon }}^{tr}+\frac{1}{2}H_{f}f^{2}+\frac{1}{2}f^{FA}H_{tw}{\tilde{\varepsilon }}^{tw}:{\tilde{\varepsilon }}^{tw}+C_{v}\left[\left(T-T_{0}\right)-T\log\frac{T}{T_{0}}\right]\\ & =U^{A}-TS^{A}+B\left(T-T_{0}\right)f-\frac{1}{2}\sigma :S:\sigma -\sigma :\alpha \Delta T-\sigma :{\tilde{\varepsilon }}^{tr}f-\sigma :{\tilde{\varepsilon }}^{tw}f^{FA}+\frac{1}{2}fH_{tr}{\tilde{\varepsilon }}^{tr}:{\tilde{\varepsilon }}^{tr}\\ & \;+\frac{1}{2}H_{f}f^{2}+\frac{1}{2}f^{FA}H_{tw}{\tilde{\varepsilon }}^{tw}:{\tilde{\varepsilon }}^{tw}+C_{v}\left[\left(T-T_{0}\right)-T\log\frac{T}{T_{0}}\right]. \end{array}$$

where $U^{A}$ and $U^{M}$ are the austenitic and the martensitic internal energy and $S^{A}$ and $S^{M}$ are the austenitic and the martensitic entropy. Several terms, such as $\Delta U=U^{M}-U^{A}$, $\Delta S=S^{M}-S^{A}$ and $\Delta T=T-T_{ref}$, are also introduced. Considering $T_{0}=\frac{\Delta U}{\Delta S}$ the equilibrium temperature of transformation, a linear variation of entropy around $T_{0}$ is defined as B=−ΔS. The terms $H_{tr}$, $H_{f}$ and $H_{tw}$ comprise, respectively, a set of material parameters characterizing interactions between grains of the microscopic RVE, between variants inside grains and between twins. $C_{v}$ is the transformation latent heat coefficient. The control equation of the thermodynamic system is established by introducing the Clausius–Duhem inequality:

(4) $$-\dot{G}-\varepsilon :\dot{\sigma }-S\dot{T}-\vec{q}\cdot \frac{\vec{grad}\;T}{T}\geq 0.$$

Substituting Equation (3) to Equation (4) and considering the thermo-elastic balance conditions, the Clausius–Duhem inequality is reduced to:

(5) $$\phi =-\frac{\partial G}{\partial f}\dot{f}-\frac{\partial G}{\partial {\tilde{\varepsilon }}^{tr}}:{\dot{\tilde{\varepsilon }}}^{tr}-\frac{\partial G}{\partial {\tilde{\varepsilon }}^{tw}}:{\dot{\tilde{\varepsilon }}}^{tw}-\vec{q}\cdot \frac{\vec{grad}\;T}{T}\geq 0.$$

Here, driving forces related to this dissipation expression are introduced:

1. Transformation driving force related to *f*:
  (6) $$\begin{array}{cc} A_{f}=-\frac{\partial G}{\partial f}= & \sigma :{\tilde{\varepsilon }}^{tr}+\zeta^{FA}\sigma :{\tilde{\varepsilon }}^{tw}-B\left(T-T_{0}\right)-\frac{1}{2}fH_{tr}{\tilde{\varepsilon }}^{tr}:{\tilde{\varepsilon }}^{tr}\\ & -H_{f}f-\frac{1}{2}\zeta^{FA}H_{tw}{\tilde{\varepsilon }}^{tw}:{\tilde{\varepsilon }}^{tw}. \end{array}$$
  where the definition of ${\dot{f}}^{FA}=\zeta^{FA}\dot{f}$ is introduced here.
2. Orientation force related to ${\tilde{\varepsilon }}^{tr}$:
  (7) $$A_{{\tilde{\varepsilon }}^{tr}}=-\frac{1}{f}\frac{\partial G}{\partial {\tilde{\varepsilon }}^{tr}}=\sigma ’-H_{tr}{\tilde{\varepsilon }}^{tr}.$$
  where σ’ denotes the deviatoric part of the stress tensor σ.
3. Twin accommodation related to ${\tilde{\varepsilon }}^{tw}$:
  (8) $$A_{{\tilde{\varepsilon }}^{tw}}=-\frac{1}{f^{FA}}\frac{\partial G}{\partial {\tilde{\varepsilon }}^{tw}}=\sigma ’-H_{tw}{\tilde{\varepsilon }}^{tw}.$$

By introducing a series of predefined critical thresholds related to these driving forces, the evolution of phase transformation is controlled. For more clarity of the relations between the main control equations, the constitutive equations for this model are summarized in Table 1.

The implementation of this model in a finite element code is realized; for more details about the implementation process, see Chemisky et al. [40] and Duval et al. [42]. Moreover, the evolution of tension-compression asymmetry and internal loops during the partial loadings can also be well simulated with this adopted model. In the following subsection, architected SMA RVEs with different geometric parameters are studied to see the cellular response of the material.

### 2.2. Convergence Analysis for the RVE Mesh

**Figure 1:**
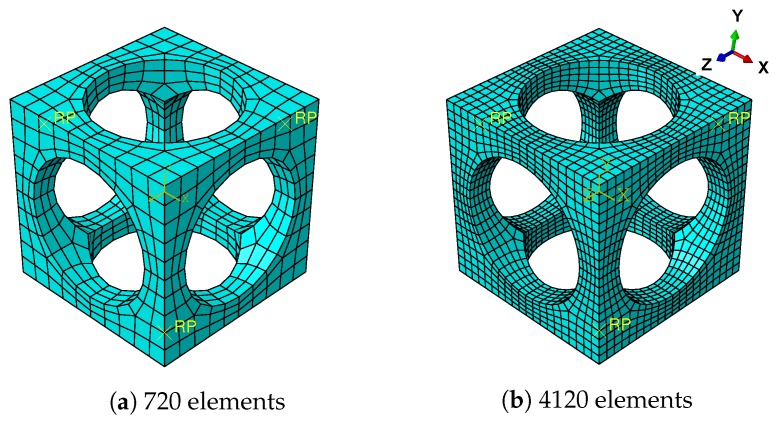
Architected RVE with fine meshes and reference points (RPs).

An architected SMA RVE is introduced to describe the microscopic structure; see Figure 1. It is formed by excavating cylindrical holes through the center of each face of a cube SMA. The size of the cube is given by 1 mm × 1 mm × 1 mm, while the radius of the cylindrical holes is given as 0.38 mm. According to computational homogenization theory, periodic boundary conditions are introduced into the RVE by the multi-point constraints (MPCs) on ABAQUS:

(9) $$\Delta u^{+}-\Delta u^{-}=\Delta \bar{\varepsilon }\cdot \left(x^{+}-x^{-}\right)\;\;on\;\;\partial \omega ,$$

where u is the displacement vector, x is the coordinates of nodes and $\bar{\varepsilon }$ is the strain load applied on the RVE. The notations + and − denote the nodes on opposite boundaries; the notation Δ represents the incremental case. Thus, a set of reference points (RPs) for three different directions is introduced in MPCs and the meshes on the opposite boundaries must be the same in order to create periodic boundaries; see Figure 1. Both the continuum 3D solid element with full integration (labeled C3D8 on ABAQUS) and continuum 3D solid element with reduced integration (labeled C3D8R on ABAQUS) for the mesh in Figure 1a are studied in order to optimize the computation efficiency on the RVE. Following the constitutive model introduced in the last subsection, the material parameters of a conventional NiTi alloy are given in Table 2, which are identified with the experimental data of Sittner et al. [43].

To check the effective response of the RVE, a tensile strain up to 10% in the Y direction is applied on the RVE at a constant temperature of 30 °C.

**Figure 2:**
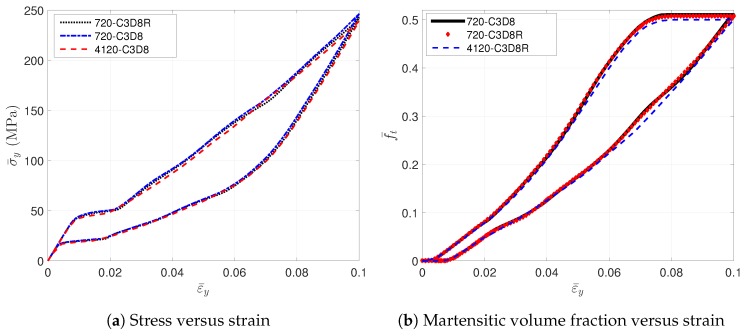
The evolution of the averaged stress and the averaged martensitic volume fraction simulated by RVEs with different meshes.

To describe the effective behavior of RVE, the averaged values, such as stress $\bar{\sigma }$, strain $\bar{\varepsilon }$ and martensitic volume fraction ${\bar{f}}_{t}$, are introduced. The averaged values are computed by volume averaging over the whole 1 mm${}^{3}$ cube from Element 1 to *N*, where *N* denotes the total element number. Note the averaged strain is equal to the prescribed strain due to the periodic boundary conditions, Equation (9). Figure 2a shows the stress-strain relations simulated with different meshes and element types. The hysteresis effect is observed during the loading/unloading cycle. This nonlinear response is caused by the inner forward phase transformation of SMA. The averaged martensitic volume fraction ${\bar{f}}_{t}$ increases along with the loads until reaching a maximum value; see Figure 2b. When the unloading begins, ${\bar{f}}_{t}$ decreases immediately until recovering to zero. From the comparison of the curves, it is observed that the mesh with 720 elements is fine enough to model this architected SMA RVE. Furthermore, the RVEs simulated by the C3D8R and C3D8 elements have exactly the same responses; see Figure 2.

Generally speaking, the mesh with 720 C3D8R elements has relatively enough accuracy and lower computational cost than other meshes. Thus, meshes like density and C3D8R elements are used for the RVE in the following work.

### 2.3. Cells with Different Geometries

**Figure 3:**
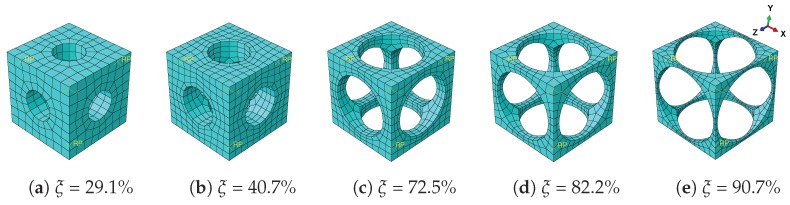
Meshes for architected SMA RVEs with different void volume fractions ξ.

In this subsection, five types of RVEs with different void volume factions ξ are studied, as illustrated in Figure 3. The size of the cube is given by 1 mm × 1 mm × 1 mm, while the radius of the cylindrical hole varying along with ξ is given in Table 3. The material parameters of SMA remain the same as those in the last subsection.

**Figure 4:**
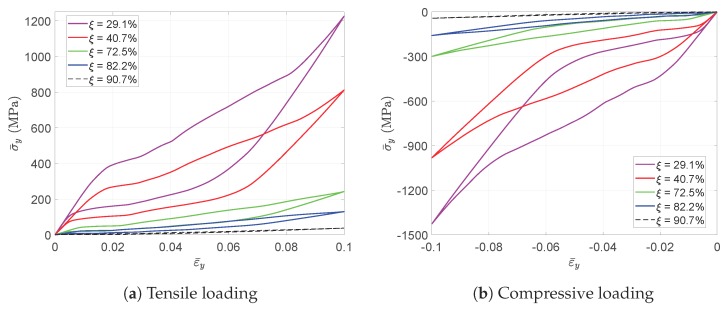
The evolution of the averaged stress-strain curves simulated by RVEs with different void fractions.

**Figure 5:**
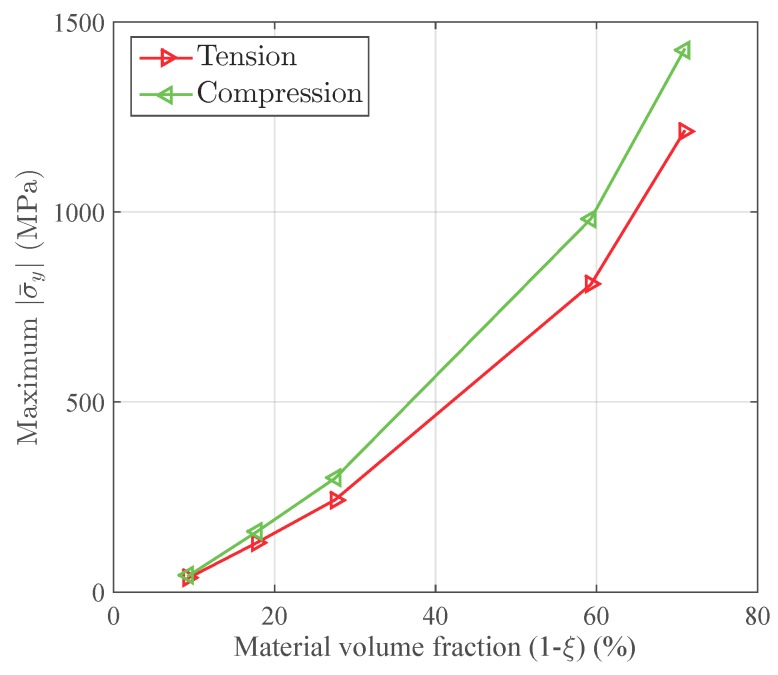
The relation between the stiffness and the material volume fraction (1-ξ) of the RVE when the absolute value of strain is up to 10%.

**Figure 6:**
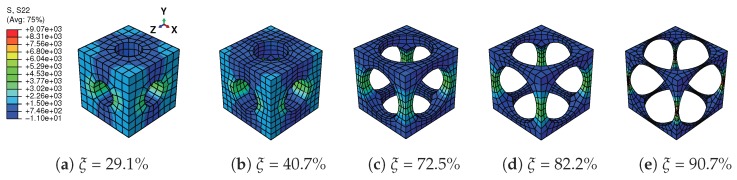
Distribution of stress in Y direction (S22) on the RVEs with different void volume fractions ξ in tension loading at strain level 10%.

**Figure 7:**
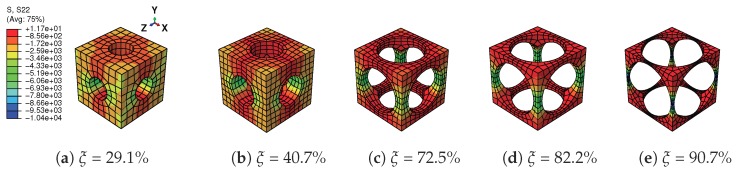
Distribution of stress in Y direction (S22) on the RVEs with different void volume fractions ξ in compression loading at strain level −10%.

A tensile strain up to 10% and a compressive strain up to −10% in the Y direction are respectively applied on the RVEs to simulate effective responses for different RVEs. Figure 4 gives the curves of averaged stress versus the averaged stain along the loading direction, simulated by the above RVEs respectively. The absolute value of stress in different RVEs at a certain strain level increases along with the decreasing of the void volume fraction, as depicted in Figure 4. In more detail, Figure 5 gives the relation of maximum stress and material volume fraction (1-ξ) when the absolute value of strain is up to 10%. The trend of the curve shows that the higher the material volume fraction is, the faster the stress increases. This trend could be explained by exploring the stress distribution on the RVE at the maximum strain level 10%, as shown in Figure 6 and Figure 7. The high stresses are mainly distributed over the middle of the pillars along the loading direction. Lower material volume fraction results in a stronger stress concentration effect. Thus, the stiffness of the RVE decreases more rapidly than the material volume fraction. This test could be a reference for designers to balance the stiffness and the mass of the architected structure.

**Figure 8:**
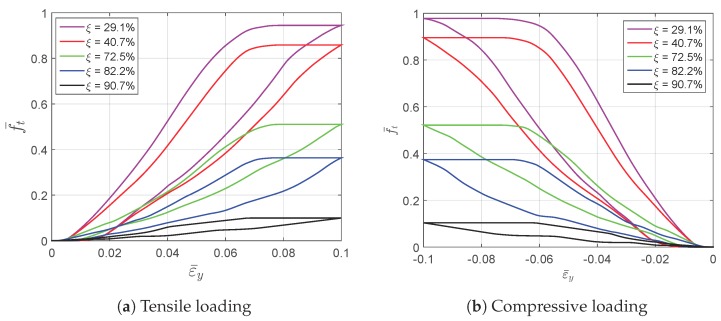
The evolution of the martensitic volume fraction versus the averaged strain curves simulated by RVEs with different void volume fractions.

**Figure 9:**
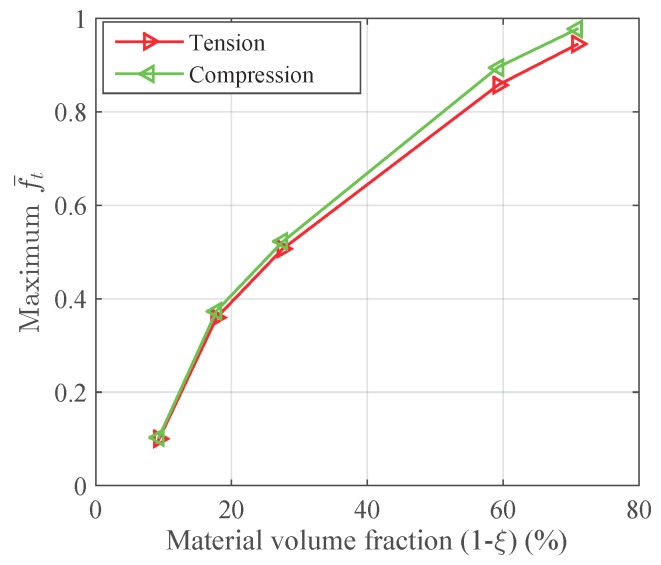
The relation between maximum martensitic volume fraction versus the material volume fraction when the absolute value of strain is up to 10%.

**Figure 10:**
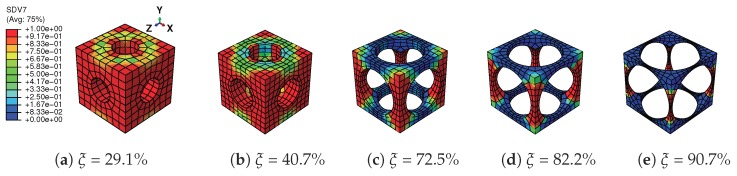
Distribution of martensitic volume fraction ${\bar{f}}_{t}$ (named SDV7 in colorbar) on the RVEs with different void volume fractions ξ in tension loading at strain level 10%.

**Figure 11:**
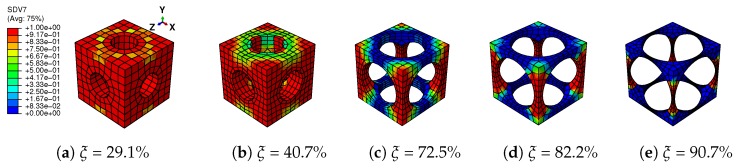
Distribution of martensitic volume fraction ${\bar{f}}_{t}$ (named SDV7 in colorbar) on the RVEs with different void volume fractions ξ in compression loading at strain level −10%.

Figure 8 gives the martensitic volume fraction averaged over SMA versus the strain averaged over the cube. The martensitic volume fraction ${\bar{f}}_{t}$ increases along with the increasing of the strain level, but stops before it reaches one. More specifically, each RVE has a certain maximum ${\bar{f}}_{t}$ value in this loading case. The maximum martensitic volume fraction versus the material volume fraction at the maximum absolute strain level of 10% is depicted in Figure 9. It shows that the lower the material volume fraction is, the more rapidly the martensitic volume fraction decreases. This also means the usage rate of SMA is at a very low level when the material volume fraction is low, and it could result in a waste of SMA. For example, the hysteresis effect of the SMA architected structure is not proportional to the SMA mass, but to the product of the SMA mass and the SMA usage rate. Thus, the hysteresis effect could be very weak when the SMA usage rate is very low. Like the stiffness discussed before, this phenomenon could also be explained by the stress concentration where only the high stress area transforms into martensite; see Figure 10 and Figure 11. This also means SMA cannot completely transform from austenite into martensite. Thus, as long as the unloading begins, the reverse transformation starts immediately, resulting in the reduction of ${\bar{f}}_{t}$.

To design a light weight architected SMA structure, it is required to minimize the material volume fraction in order to decrease the weight and cost of the structure. However, the necessary stiffness of the structure should be firstly satisfied. It should be noticed that the waste of SMA should also be avoided as much as possible due to SMA’s high price. Therefore, the results given in Figure 5 and Figure 9 can be a reference for the designers to balance the stiffness, mass and SMA usage according to their specific requirements.

Following the tests at the RVE level, the structural response of the architected SMAs is studied in the next section.

## 3. Structural Response

In this section, the structural responses of the architected SMAs are focused on. The constitutive behavior of the macroscopic structure is represented by the computational homogenized RVEs in the last subsection. To simulate the structural response, a short introduction of the multilevel finite element method (FE${}^{2}$) is presented firstly.

### 3.1. FE${}^{2}$ Formulation

**Figure 12:**
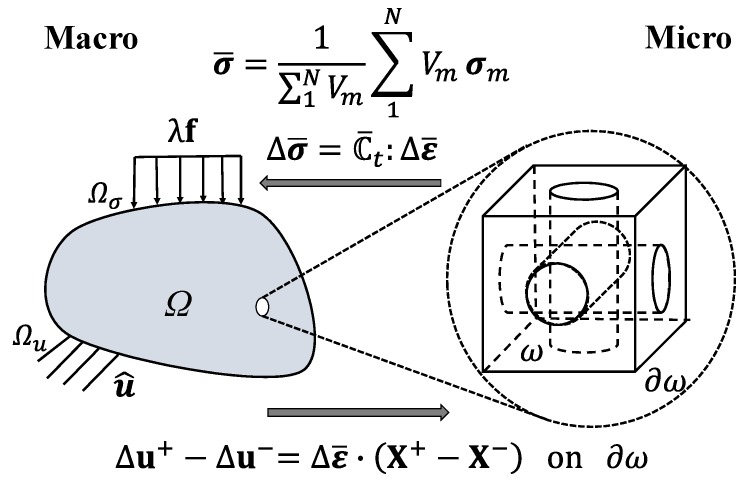
Basic concept of the finite element squared method.

Generally speaking, an architected SMA with a periodic microscopic structure can be divided into two scales and be simulated by the multiscale homogenization method. As depicted in Figure 12, each point at the macroscopic level is represented by a periodic RVE after homogenization. The constitutive behavior and the stress of each macroscopic point are transferred from the RVE by the computational homogenization technique, while the macroscopic strain is applied on the RVE with periodic boundary conditions. Both levels are simulated by FEM, which can capture their mechanical fields accurately. This approach is implemented on ABAQUS via the user subroutine UMAT. The real-time interaction of the two levels is realized by the iteration of the Newton–Raphson method. This method is able to compute both the macroscopic and microscopic responses of the structures simultaneously. The authors have developed this FE${}^{2}$ approach on the ABAQUS platform for SMA-based composites; see Xu et al. [39] for more detailed formulations. Related valuable works on multiscale modeling of SMA composite could be also referred to, such as Kohlhaas and Klinkel [37], Chatzigeorgiou et al. [38], Chatzigeorgiou et al. [44] and Fatemi Dehaghani et al. [45].

### 3.2. Beam with Three Kinds of Cells Subjected to Three-Point Bending

**Figure 13:**
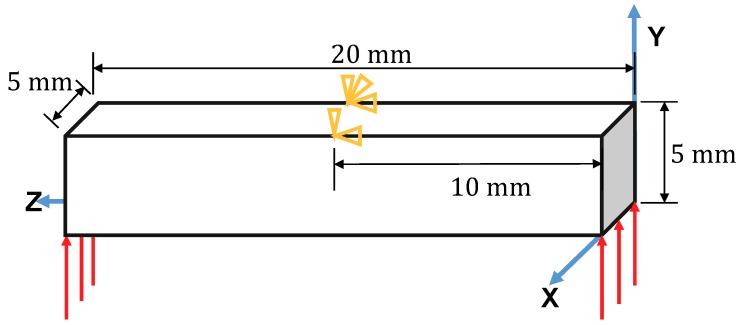
Geometry and boundary conditions for the three-point bending beam.

The multiscale finite element method mentioned above has been validated by the reference and shows good ability in modeling the superelasticity and the shape memory effect of the SMA composites [39]. Therefore, this multiscale model is used to simulate the multiscale response of the architected SMA structure herein. A 3D beam subjected to three-point bending load is shown in Figure 13.

**Figure 14:**
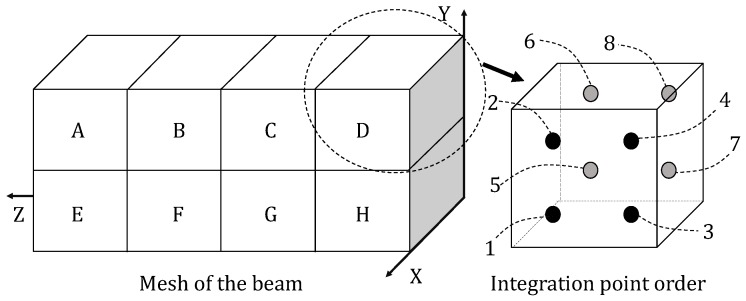
The meshes for the left half beam and the integration points in each C3D8I element.

This beam is composed of architected SMA. The width, length and height of the beam are 5 mm, 20 mm and 5 mm, respectively. Edge Y = 5 mm, Z = 10 mm is fixed in the Y and Z directions. A displacement load up to 0.5 mm in the Y direction is applied on the edges Y = Z = 0 mm and Z = 20 mm, Y = 0 mm. Node X = 0 mm, Y = 5 mm, Z = 10 mm is fixed in the X direction in order to eliminate the rigid body displacement in the X direction. Considering the symmetry of the structure and the boundaries, only the left half of the structure is simulated in order to reduce the computation cost. To do this, an additional displacement constraint in the Z direction is given on face Z = 10 mm. Since the deformation in the X direction is not obvious in the three-point bending test, one element is used in this direction. Each edge in the Y direction is meshed by two elements and in the Z direction by four elements; see Figure 14. The continuum 3D solid element with incompatible modes (labeled C3D8I in ABAQUS) is adopted for the modeling of the macroscopic beam since it is enhanced by incompatible modes to improve its bending behavior. The RVEs studied in the last section are used herein with void volume fractions ξ of 40.7%, 72.5% and 82.2%, respectively.

**Figure 15:**
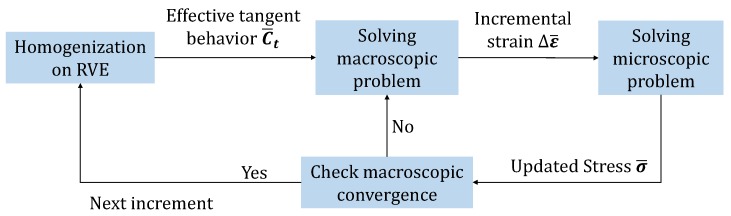
The nonlinear interaction between two scales in the Newton–Raphson framework.

As the macroscopic constitutive behavior on each integration point is not clear, it has to be represented by the effective behavior of the associated RVE at each macroscopic increment. A brief flow diagram, showing how this multiscale problem is solved, is illustrated in Figure 15. Specifically, the effective behavior of the RVE is computed by seeking the relations between the averaged stresses and averaged strains over the RVE via a series of loading tests. Once the effective behavior is obtained, the macroscopic problem is to be solved. Considering the nonlinear response of the RVE during loading, the macroscopic convergence has to be checked in each macroscopic iteration of the Newton–Raphson framework. In an iteration, the strain states of RVEs are updated with the macroscopic strains, and in return, the macroscopic stresses are renewed by updating the averaged stress of the RVE at the new strain states.

### 3.3. Stress Distributions at the Macroscopic and Microscopic Levels

**Figure 16:**
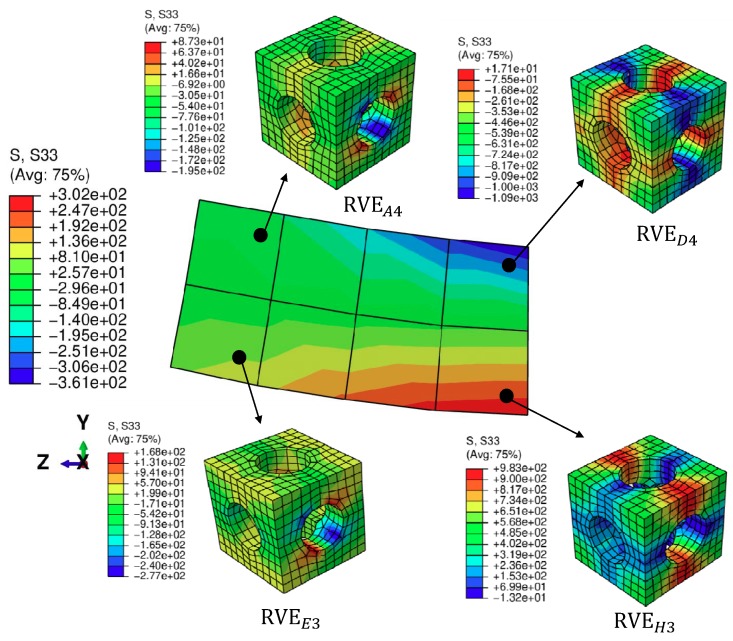
The distribution of stress in Z direction (S33) for the macroscopic structure and microscopic structure with ξ = 40.7%, where the deformations of both levels are magnified by three times.

Let us consider the case with volume fraction ξ = 40.7%. The stress distribution of the bending beam with the prescribed displacement in the Y direction reaching 0.5 mm is illustrated in Figure 16. The deformations of both the macroscopic and microscopic structures are magnified by three times. The high compressive stresses in the Z direction are observed in the elements above the middle plane Y = 2.5 mm, while in contrast, the compressive stresses are observed in the elements below the middle plane. In the meantime, for different macroscopic points, the RVEs have different stress states in correspondence with their associated macroscopic strain states. To specify the microscopic structure more clearly, we introduce RVE${}_{kl}$ to denote an RVE corresponding to the integration point *l* of the macroscopic element *k*. The RVEs above the middle plane, such as RVE${}_{A4}$ and RVE${}_{D4}$, are mainly subjected to compression, while the RVEs below the middle plane, such as RVE${}_{E3}$ and RVE${}_{H3}$, are mainly subjected to tension. The stresses in the RVEs far from face Z = 10 mm, such as RVE${}_{A4}$ and RVE${}_{E3}$, are at a relatively low level compared to those RVEs near face Z = 10 mm, such as RVE${}_{D4}$ and RVE${}_{H3}$.

### 3.4. Evolution of the Loading

**Figure 17:**
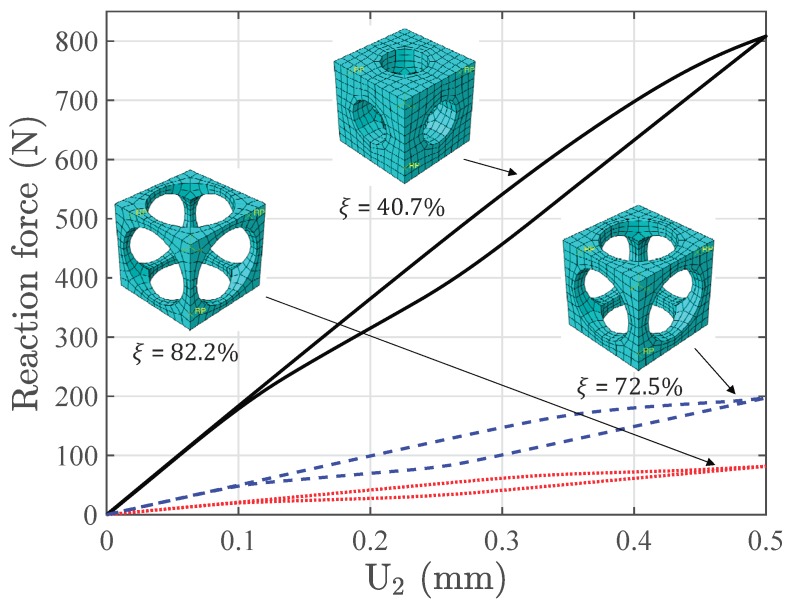
Force-displacement curve of the boundary Z = 20 mm, Y = 0 mm on the bending beam.

**Figure 18:**
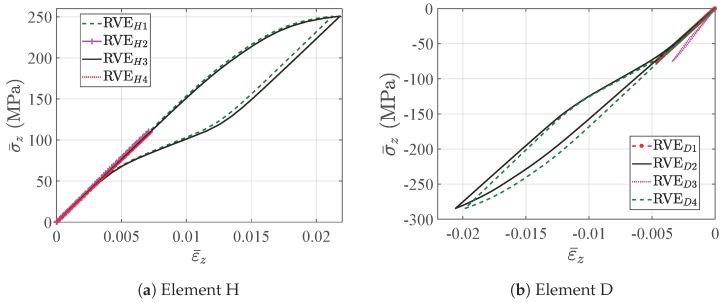
The stress-strain relations on the macroscopic integration points with ξ = 40.7%.

**Figure 19:**
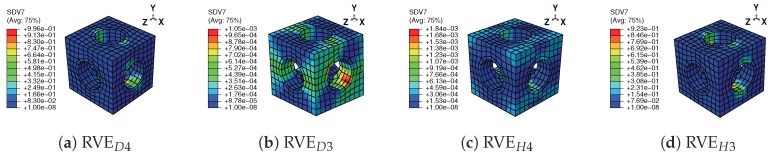
Distribution of martensitic volume fraction ${\bar{f}}_{t}$ (named SDV7 in colorbar) on the RVEs with the prescribed displacement in the Y direction reaching 0.5 mm and ξ = 40.7%.

Figure 17 shows the nonlinear response of the macroscopic structure with three different void fractions. The linear response is observed at the very beginning, and the microscopic structures deform without any phase transformation. As the loading increases, the forward phase transformation begins over the high stress area of the beam. For example, Figure 18 gives the stress strain relations of integration points in element D and element H with ξ = 40.7%, which also represent the effective behavior of the associated RVEs. Note that Integration Points 5 to 8 in each element are not illustrated considering the symmetry of the structure in the middle plane X = 2.5 mm. It is observed that the hysteresis effects in RVE${}_{D3}$ and RVE${}_{H3}$ are relatively much more obvious than those in RVE${}_{D4}$ and RVE${}_{H4}$; because RVE${}_{D3}$ and RVE${}_{H3}$ are located in the high stress area. The martensite transformation states of the RVEs with the prescribed displacement in the Y direction reaching 0.5 mm, shown in Figure 19, also give a reasonable confirmation from the RVE level. This figure depicts the RVEs corresponding to these integration points closest to the face Z = 10 mm. Only the RVEs far from the middle plane Y = 2.5 mm have an obvious phase transformation. Simultaneously, the phase transformation at the RVE level is accompanied by the softening of macroscopic stiffness. This softening goes gradually, because the thermomechanical phase transformations at the microscopic level are not synchronous, considering that the stress states of the macroscopic structure are not uniform over the whole beam and that the microscopic structure is architected. Once the unloading begins, the behaviors of the structure and RVEs immediately turn into the linear case. Later, the reverse transformation follows, and finally, all the phases transform back into austenite.

### 3.5. Structural Response with Different Microscopic Structures

**Figure 20:**
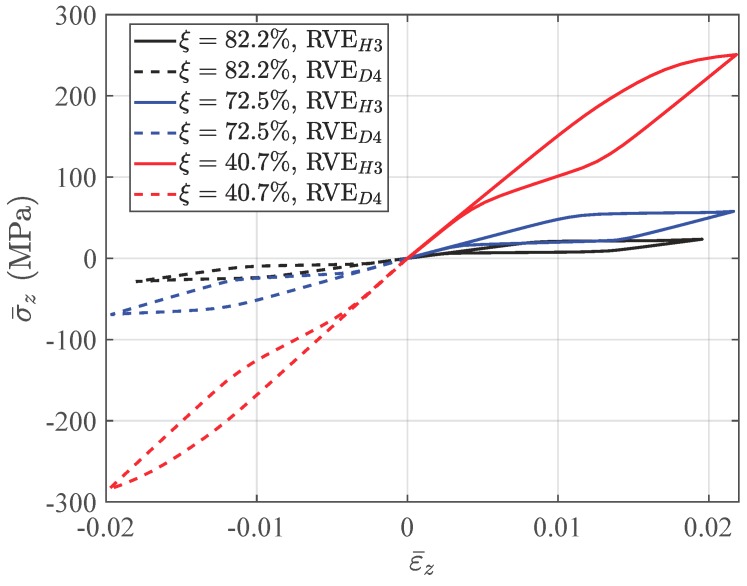
The comparison of the stress-strain relations on the macroscopic Integration Points *H3* and *D4* simulated by three kinds of RVEs respectively.

**Figure 21:**
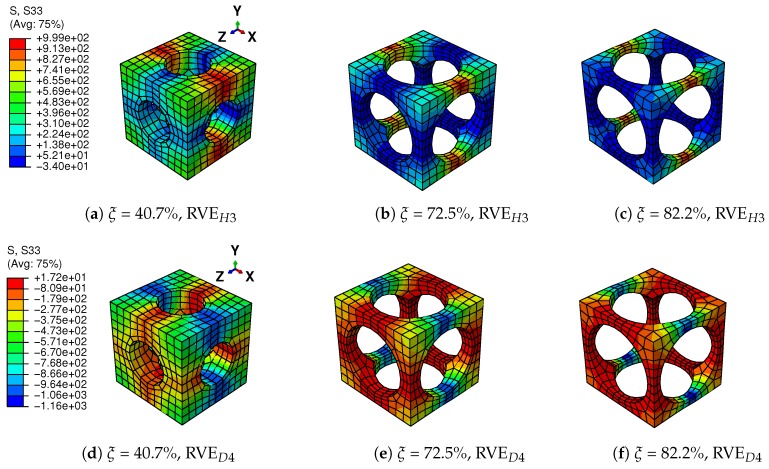
Distribution of stress in Z direction (S33) on RVE${}_{H3}$ and RVE${}_{D4}$ of different beams corresponding to the displacement of the beam ends U${}_{2}$ = 0.5 mm.

The differences between structural responses with three void fractions are observed in Figure 17. With a higher void fraction, the macroscopic structure shows lower stiffness and a weaker hysteresis effect, because the macroscopic behavior is represented by the mean behavior of the microscopic structures. The stress-strain relations of Integration Points *H3* and *D4*, which are also the averaged stress-strain relations of RVE${}_{H3}$ and RVE${}_{D4}$, are depicted in Figure 20 to investigate the microscopic response of the structure. Figure 21 gives the stress distributions in the Z direction for associated RVEs at maximum loads corresponding to the displacement of the beam ends U${}_{2}$ = 0.5 mm. It is observed that the averaged stiffness and the hysteresis effect of the RVE with a higher void fraction are lower than those of the RVE with a lower void fraction, which is consistent with the response at the macroscopic level. As we have discussed in Section 2, the high void fraction results in less SMA, which can provide low stiffness for the structure. For this architected structure, the stresses are concentrated over the pillars along the main loading direction when the void fraction is high. This also results in the martensite phase being mainly concentrated over the pillars.

### 3.6. Comments on the Computational Efficiency

The proposed numerical tool contributes to the design of innovative SMA applications, which can optimize the quantity of material with the required functionalities (actuation, recovery force, damping, energy absorption, etc.), lighten the structure and consider alternative manufacturing processes such as additive manufacturing. The latter can consider more complex geometries of the unit cell, which has a direct influence on the overall behavior of the structure. Note that this numerical multiscale procedure is very time consuming. To reduce the CPU time, the authors propose to associate this method with model reduction techniques. Among these techniques, we can introduce the proper orthogonal decomposition (POD) to build up a small number of basis functions where the solution can be computed (Yvonnet et al. [46]). When this technique is used in an optimization procedure requiring several repetitive calculations, it can significantly reduce the computation time. We can also consider the proper generalized decomposition (PGD) technique (Ammar et al. [47]; Kpogan et al. [48]), which can reduce the dimensionality of the problem to be solved and which is well adapted for unsteady problems, as well as parametric studies. Another technique that has proven its efficiency in solving non-linear problems and that was developed by our group is the asymptotic numerical method (ANM). This technique is based on the development of variables in the form of Taylor series truncated at large order, which allows one to reduce the computation time significantly by reducing the number of computation steps. This technique can be considered as a high order predictor algorithm without the need for any iteration phase. It is particularly suited for strong nonlinearities and for thin-structure problems involving instability phenomena (Nezamabadi et al. [49]; Assidi et al. [50]; Aggoune et al. [51]). In addition, bridging techniques (Hu et al. [52]; Yu et al. [53]) can also be considered to combine the advantages of different reduced finite element models. These tools will be associated with the present algorithm to reduce the computation time and to deal with complex structural geometry and complex response curves.

## 4. Conclusions

In this paper, the superelasticity behavior of the architected SMA structure is studied with a 3D multiscale finite element model. Firstly, RVEs with architected SMA are built to simulate the cellular responses of the structure. The behavior of SMA is described by the constitutive model proposed by Chemisky et al. [40]. Both tension and compression loading cycles are applied on the RVEs with different void fractions. The superelasticity responses and the hysteresis effects are observed in the RVEs. The effect of changing the void fraction on the stiffness, the maximum martensitic volume fraction and hysteresis effect are discussed in detail. Moreover, a multiscale approach is carried out to model the structural response, as well as the cellular response. The relations between the structural and cellular responses are studied in a three-point bending test. It is observed that the macroscopic response is related to the phase transformation in the RVE, which changes the effective constitutive behavior of the structure. The stress-strain state of the RVE directly depends on the stress strain state of the associated macroscopic point. Thus, the multiscale model is necessary and successful for simulating the nonlinear behavior of the architected SMA structure. Furthermore, structural responses with different void fractions are studied, which gives a good reference of the void fractions’ influences on structural stiffness and hysteresis.

## Figures and Tables

**Table 1:** Summary of the control equations for the SMA model.

Strain mechanisms:
$\varepsilon =\varepsilon^{e}+\varepsilon^{th}+\varepsilon^{tr}+\varepsilon^{tw}.$
Thermodynamical potential:
$G=U^{A}-TS^{A}+B\left(T-T_{0}\right)f-\frac{1}{2}\sigma :S:\sigma -\sigma :\alpha \Delta T-\sigma :{\tilde{\varepsilon }}^{tr}f-\sigma :{\tilde{\varepsilon }}^{tw}f^{FA}+\frac{1}{2}fH_{tr}{\tilde{\varepsilon }}^{tr}:{\tilde{\varepsilon }}^{tr}+\frac{1}{2}H_{f}f^{2}+\frac{1}{2}f^{FA}H_{tw}{\tilde{\varepsilon }}^{tw}:{\tilde{\varepsilon }}^{tw}+C_{v}\left[\left(T-T_{0}\right)-T\log\frac{T}{T_{0}}\right].$
Clausius–Duhem inequality:
$-\dot{G}-\varepsilon :\dot{\sigma }-S\dot{T}-\vec{q}\cdot \frac{\vec{grad}\;T}{T}\geq 0.$
Thermo-elastic balance conditions:
$S:\sigma +\alpha \Delta T+f{\tilde{\varepsilon }}^{tr}+f^{FA}{\tilde{\varepsilon }}^{tw}-\varepsilon =0,$
$S^{A}-Bf+\sigma :\alpha -C_{v}\left(\log\frac{T}{T_{0}}\right)-S=0.$
Thermodynamic forces:
$A_{f}=-\frac{\partial G}{\partial f}=\sigma :{\tilde{\varepsilon }}^{tr}+\zeta^{FA}\sigma :{\tilde{\varepsilon }}^{tw}-B\left(T-T_{0}\right)-\frac{1}{2}fH_{tr}{\tilde{\varepsilon }}^{tr}:{\tilde{\varepsilon }}^{tr}-H_{f}f-\frac{1}{2}\zeta^{FA}H_{tw}{\tilde{\varepsilon }}^{tw}:{\tilde{\varepsilon }}^{tw},$
$A_{{\tilde{\varepsilon }}^{tr}}=-\frac{1}{f}\frac{\partial G}{\partial {\tilde{\varepsilon }}^{tr}}=\sigma ’-H_{tr}{\tilde{\varepsilon }}^{tr},$
$A_{{\tilde{\varepsilon }}^{tw}}=-\frac{1}{f^{FA}}\frac{\partial G}{\partial {\tilde{\varepsilon }}^{tw}}=\sigma ’-H_{tw}{\tilde{\varepsilon }}^{tw}.$
Criterion functions:
$F_{f}^{crit}=F_{f}^{max}+\left(B_{f}-B\right)\cdot \left(T-T_{0}\right)\;if\;\dot{f}>0,$
$F_{f}^{crit}=-F_{f}^{max}+\left(B_{r}-B\right)\cdot \left(T-T_{0}\right)\;if\;\dot{f}<0.$
Physical limitations:
0≤f≤1.

**Table 2:** Material parameters for SMA.

*E* (MPa)	39,500	$B_{r}$ (MPa °C${}^{-1}$)	7	$H_{f}$ (MPa)	2
ν	0.3	$B_{f}$ (MPa °C${}^{-1}$)	7	$H_{tr}$ (MPa)	1635
$\varepsilon_{trac}^{T}$	0.056	$M_{s}$ (°C)	−80	$H_{tw}$ (MPa)	25,000
$\varepsilon_{trac}^{TFA}$	0.053	$A_{f}$ (°C)	−2	$H_{s}$ (MPa)	68.5
$\varepsilon_{comp}^{T}$	0.044	$F_{\varepsilon }$ (MPa)	220		

**Table 3:** Geometrical parameters for RVEs.

Radius (mm)	0.2	0.24	0.38	0.42	0.47
Voidvolumefractionξ	29.1%	40.7%	72.5%	82.2%	90.7%

## Abbreviations

The following abbreviations are used in this manuscript:

SMA	Shape memory alloy
RVE	Representative volume element
FE${}^{2}$	Multilevel finite element method
FEM	Finite element method
UMAT	User-defined materials
MPCs	Multi-point constraints
RP	Reference point
C3D8	Continuum 3D solid element with full integration
C3D8R	Continuum 3D solid element with reduced integration
C3D8I	Continuum 3D solid element with incompatible modes
